# Enabling Stable Recycling of L-Arabinose Isomerase Through Whole-Cell Immobilization for Efficient and Cost-Effective D-Tagatose Production

**DOI:** 10.3390/foods14091538

**Published:** 2025-04-28

**Authors:** Zepeng Li, Runmin Wang, Xiantai Lai, Wenyi Liao, Runfeng Liao, Zhuohong Wu, Guoyan Zhang, Xianghui Qi

**Affiliations:** School of Life Sciences, Guangzhou University, Guangzhou 510006, China; 32214130043@e.gzhu.edu.cn (Z.L.); 32214130001@e.gzhu.edu.cn (R.W.); 32314130014@e.gzhu.edu.cn (X.L.); 32210100026@e.gzhu.edu.cn (W.L.); 32214130025@e.gzhu.edu.cn (R.L.); 32214130033@e.gzhu.edu.cn (Z.W.)

**Keywords:** D-tagatose, L-arabinose isomerase, immobilized cells, alginate, stability

## Abstract

D-tagatose is a functional sweetener with glucose-regulating and prebiotic properties, but its bioproduction from D-galactose faces many limitations, particularly the high production costs. In particular, the current biosynthesis of D-tagatose suffers from thermal instability and the substrate selectivity issues of L-arabinose isomerase (L-AI) required to convert D-galactose into D-tagatose. In this study, recombinant *Escherichia coli* BW25113/pQE-80L-*araA*_F118M/F279I_ expressing double mutant L-AI was immobilized to enhance its stability and reusability. The optimal conditions for whole-cell catalysis were 60 °C, pH 6.5, 5 mM Mn^2+^, and 20 h, with a yield of 55.2 g/L of D-tagatose. Immobilization with 3% sodium alginate and 2% CaCl_2_ retained 90% of the production efficiency displayed by free cells. Notably, the immobilized cells exhibited enhanced heat resistance (60–70 °C) and operational stability, retaining 76% activity after five cycles. The D-tagatose production was further increased to 129.43 g/L by increasing the substrate concentration to 250 g/L. Compared to free cells, immobilized cells retained 83.6% of the initial yield up to 10 batches. This study presents a cost-effective and sustainable method for the production of D-tagatose using optimized whole-cell catalysis through immobilization, which paves the way to solve industrial challenges such as thermal instability and low substrate efficiency.

## 1. Introduction

With a rise in the prevalence of metabolic syndrome globally, the chronic health risks associated with excessive intake of traditional sweeteners have emerged as a significant public health concern [1]. For example, a recent study found a significant and positive correlation between added sugar intake and the prevalence of diabetes (8%), obesity (12%), and hypertension (47%) among Gambian adults [2]. Therefore, traditional sweeteners that are high in fructose and sucrose are gradually being replaced by rare sugars due to their low caloric value [3,4]. D-tagatose is one of the most common rare sugars. As a natural six-carbon keto sugar, D-Tagatose not only possesses a similar taste of sweetness (about 92% relative sweetness) to sucrose but also has a calorie content of only 1.5 kcal/g (one-third of sucrose), demonstrating significant nutritional advantages and broad application prospects [5,6]. In 2001, the U.S. Food and Drug Administration (FDA) included D-tagatose in the list of Generally Recognized as Safe (GRAS) substances [7]. Notably, China approved D-tagatose as a novel food ingredient in the Food Safety Law of the People’s Republic of China and Administrative Measures for the Safety Review of New Food Ingredients in 2014 [8]. D-tagatose inhibits small intestinal glucose transport and hepatic glucose metabolism, thereby lowering postprandial blood glucose peaks, while it increases red blood cell counts, coagulation factors attenuating oxidative stress (21%), and expression of the inflammatory factors [9,10]. In addition, there are favorable benefits in gut health regulation (selective promotion of probiotics and colonocytes) and oral health (blocking cariogenic bacteria) [11,12]. Based on these properties, D-tagatose has been widely used in functional food fields, complementary therapeutic drugs, and cosmetics. It is suitable for use in bakery and cereal products due to its ability to produce volatile flavor substances (2-ethylpyrazine) and low hygroscopicity [13]. As a low-calorie sweetener, it can be used in carbonated beverages to improve the mouthfeel of the beverage, and in chocolate products to reduce the calorie content [14]. In addition, it can be added to meat products to inhibit microbial spoilage and extend shelf life [15]. In the pharmaceutical field, D-tagatose is used as an excipient and dietary supplement for the treatment of diabetes, obesity, and constipation (e.g., the drug Chronulac^®^) [16].

Current industrial production of D-tagatose relies on enzymatic isomerization, where D-galactose is converted to D-tagatose by L-arabinose isomerase (L-AI). This biological method has received much attention from researchers because of its significant advantages over chemical synthesis routes, such as being green, mild, and sustainable [17]. On the other hand, Carla et al. attempted to produce D-tagatose from sucrose present in a dairy by-product named whey powder with a tagatose yield of 23% by utilizing β-Galactosidase (β-GAL) from *Aspergillus oryzae* and L-AI from *Thermotoga maritima*, which provided a more sustained option [18]. However, the low substrate specificity of native L-AI for D-galactose, lack of thermal stability, and high production costs limit its industrial feasibility. L-AIs from mesophilic microorganisms usually operate optimally at temperatures lower than 50 °C, whereas industrial processes require temperatures above 60 °C to improve reaction rates and product yields [19]. In addition, free enzymes are rapidly inactivated at high temperatures, requiring repeated purification and high operating costs. Although genetic engineering has improved catalytic efficiency through rational/semi-rational design, the resulting enzymes often exhibit impaired thermal stability [20]. The incorporation of non-natural amino acids into L-AI has shown promise in stabilizing proteins, but their application remains limited due to technical complexity and cost constraints [21].

To improve stability, immobilization of whole cells or enzymes is widely used in industrial bioprocessing. Compared to soluble enzymes, immobilized systems offer the advantages of easy reusability, greater stability under harsh conditions, and simplified downstream processing [22]. Shushil et al. self-assembled a hybrid nanofluid (MnNF@L-AI) using manganese phosphates and *Lactobacillus sakai* L-AI (LSAI) with an optimal temperature increased by 10 °C compared to free LSAI and was able to maintain a higher activity than LSAI. In addition to improved stability, MnNF@L-AI showed excellent reusability [23]. Torres et al. immobilized the triple-enzyme system of β-GAL, D-xylulose isomerase (D-GI), and L-AI on ethylene oxide-carrying Eupergit resin for the production of D-tagatose from whey via the immobilizers, and the immobilized reactors showed improved performance compared to soluble enzymes [24]. In addition, alginate microspheres crosslinked with glutaraldehyde also exhibited better thermal and operational stability than free enzyme/cells in the immobilization of L-AI in both enzymes and cells [25,26,27].

Based on the above facts, this study focuses on immobilizing the *Escherichia coli* recombinant strain which overexpressing *araA*_F118M/F279I_ mutant enzyme [28], using sodium alginate. Firstly, the biochemical reaction conditions of free whole cells, including temperature, metal ions, pH value, and reaction time, were optimized. Then, the key parameters of immobilization and embedding process were studied to enhance cell stability and efficiency. Finally, batch replenishment transformation was conducted to compare the conversion efficiency and reusability of the immobilized system with free enzymes at elevated temperatures. In this way, the use of sodium alginate immobilized cells provides a more stable and cost-effective method for the production of D-tagatose, which can maintain the activity for a longer period of time at higher temperatures and is conducive to the continuous and efficient production of D-tagatose. This innovative approach overcomes key limitations in conventional free-cell systems, offering a practical solution for industrial-scale D-tagatose manufacturing with reduced biocatalyst costs and improved process sustainability.

## 2. Materials and Methods

### 2.1. Strains, Culture, and Reagents

The strain *E. coli* BW25113/pQE-80L-*araA*_F118M/F279I_, harboring the recombinant plasmid pQE-80L-*araA*_F118M/F2791_ with LpAI double-point mutation, was constructed in our previous study, and designated as EC*araA*_F118M/F279I_ [28]. The strain was cultured in LB medium (10 g/L tryptone, 5 g/L yeast extract, and 10 g/L NaCl) at 37 °C with shaking at 220 rpm. Standards of D-galactose, D-tagatose, and isopropyl-β-D-thiogalactoside (IPTG) were purchased from Shanghai Aladdin Biochemical Technology Co., Ltd. (Shanghai, China). Ampicillin (Amp) was obtained from Beijing Solebo Technology Co., Ltd., Beijing, China. D-galactose, Triton-x-100, acetic acid, sodium acetate, sodium alginate, anhydrous Calcium chloride, Sodium Chloride, Manganese sulfate (Beijing, China), and other chemicals/reagents were purchased from Sinopharm Chemical Reagent Co., Ltd. (Shanghai, China).

### 2.2. Optimal Reaction Conditions of Whole-Cell ECaraA_F118M/F279I_

#### 2.2.1. Preparation of Whole-Cell EC*araA*_F118M/F279I_

The recombinant strain EC*araA*_F118M/F279I_ was cultured overnight at 37 °C on the LB-Amp plate. The single colony was transferred to 5 mL LB-Amp liquid medium and incubated at 37 °C, 220 rpm to prepare the seed culture. Subsequently, 1% (*v*/*v*) of the seed culture was transferred to a 200 mL LB-Amp liquid medium. When OD_600_ was reached from 0.6 to 0.8, IPTG was added to a final concentration of 0.1 mM to induce the expression of the *araA*_F118M/F279I_. Induction was carried out at 20 °C and 120 rpm for 12 h. After that, the bacterial cells were harvested by centrifugation at 6000 rpm for 10 min at 4 °C. The cell pellet was resuspended in PBS (pH 7.4) containing 2% Triton-x-100 and incubated at room temperature for 30 min. The cells were centrifuged at 6000 rpm for 10 min at 4 °C, and the supernatant was discarded. Finally, the cell pellet was suspended and washed 3 times with PBS (pH 7.4) and was then re-suspended in PBS (pH 7.4) for further enzymatic assays.

#### 2.2.2. Determination of Optimal Reaction Conditions

Firstly, the whole cells with a wet weight of 100 g/L were mixed with 150 g/L D-galactose solution containing 1–10 mM Mn^2+^ (pH 6.0). Then, whole cells with a wet weight of 100 g/L were incubated in 150 g/L D-galactose solution at pH 6.0 and various temperatures, such as 45 °C, 50 °C, 55 °C, 60 °C, and 65 °C, for 2 h to determine the optimum reaction temperature. After that, substrate solutions with varying pH values were prepared using 10 mM acetate buffer (pH 5.0–6.0) and 10 mM phosphate buffer (pH 6.5–8.0), with pH values adjusted to 5.0, 5.5, 6.0, 6.5, 7.0, 7.5, and 8.0, respectively, to determine the optimal reaction pH. All above optimization experiments were conducted in 5 mL reaction volumes with 2 h incubation at 220 rpm. Following this, the reaction time was evaluated under the optimum Mn^2+^, temperature, and pH. Aliquots of 1 mL were collected at intervals of 1 h, 5 h, 10 h, 20 h, 48 h, 72 h, and 96 h, and the titer of D-tagatose was quantified to determine the reaction time of the whole-cell biocatalyst. After the incubation period, the reaction was terminated by immersing the mixture in a boiling water bath for 10 min to ensure complete deactivation of the cells. The titer of D-tagatose was measured by HPLC, and the enzyme activity was calculated to determine the optimum concentration of Mn^2+^ for the reaction. The maximum enzyme activity was defined as 100%, and the relative enzyme activities at other assays were expressed as percentages relative to this maximum value.

### 2.3. Preparation of Immobilized Cell Catalyst and Biocatalyst Activity

The whole cells of EC*araA*_F118M/F279I_ were prepared as described in Section 2.2.1, with the addition of 5 mM Mn^2+^ for subsequent cell immobilization. A suitable concentration (1~5%) of sodium alginate was dissolved in 10 mM acetate buffer (pH 6.5). An appropriate number of whole cells was then added to the sodium alginate solution and mixed thoroughly by stirring for 10–20 min. The resulting mixture was slowly dripped into a CaCl_2_ solution (pH 6.5) using the disposable syringe to form uniform immobilized beads. The beads were washed three times with 10 mM acetate buffer (pH 6. 5) and stored at 4 °C for further use. For the biocatalysis reaction, 5 mL of 150 g/L D-galactose solution containing 5 mM Mn^2+^ was prepared in 10 mM acetate buffer (pH 6.5). Free cells and immobilized cells were added separately. The reaction mixtures were incubated at 60 °C with shaking at 220 rpm for 5 h, after which the reactions were terminated in a boiling water bath for 10 min. The enzymatic activity of both free and immobilized cells was determined by quantifying D-tagatose production via HPLC. Enzyme activity units (U) were defined as the amount of catalyst required to produce 1 μmol of D-tagatose per minute under different assay conditions. For comparative analysis, relative activity (%) was calculated by normalizing all values to the maximum observed activity (set as 100%) at different trails.

### 2.4. Optimization of Immobilized Cells Preparation

A substrate solution containing 150 g/L D-galactose and 5 mM Mn^2+^ was prepared, and key parameters, including CaCl_2_ concentration, sodium alginate concentration, hardening time, and wet cell concentration, were optimized using a single-factor optimization strategy. To determine the optimal CaCl_2_ concentration, whole cells mixed with 2% sodium alginate were dripped into CaCl_2_ solution (pH 6.5) with concentrations of 2%, 4%, 6%, 8%, and 10%, respectively, and 0.2 g of immobilized cells from each group was added to the substrate solution. To optimize the alginate concentration, sodium alginate solutions (1, 2, 3, 4, and 5%, pH 6.5) were prepared and thoroughly mixed with 100 g/L wet whole cells. To evaluate the hardening time, the prepared beads were allowed to harden at room temperature for 2 h, 4 h, 6 h, 8 h, and 10 h, respectively. For determining the optimal cell loading, cell concentrations of 20 g/L, 40 g/L, 60 g/L, 80 g/L, 100 g/L, 120 g/L, 160 g/L, and 200 g/L were mixed with sodium alginate solution (pH 6.5). All reactions were incubated at 60 °C with shaking at 220 rpm for 2 h. The titer of D-tagatose was quantified to determine the enzyme activity of the immobilized cells in each group.

### 2.5. Optimization of Reaction Conditions and Immobilized Cells Stability Accessment

To determine the optimal reaction temperature, 0.2 g of immobilized cells was added to the substrate solution (prepared as described in Section 2.2.2) and incubated at 45 °C, 50 °C, 55 °C, 60 °C, and 65 °C for 2 h and 220 rpm, respectively. To evaluate the optimal reaction pH, substrate solutions with varying pH values (prepared as outlined in Section 2.2.2) were incubated with 0.2 g immobilized cells at the previously determined optimal temperature for 2 h and 220 rpm. For thermal stability assessment, 100 g/L free cells and 0.5 g immobilized cells were separately added to the substrate solution and subjected to six consecutive reaction cycles (2 h per cycle) at 60 °C, 65 °C, and 70 °C. After each cycle, both free and immobilized cells were washed with 10 mM acetate buffer (pH 6.5) to remove residual substrates and re-equilibrated in a fresh substrate solution of identical composition. 0.2 g of immobilized cells were reacted in a water bath at the optimal temperature for 96 h. Changes in the trend of D-galactose conversion of the immobilized cells were analyzed at intervals of 1, 5, 10, 20, 48, 72, and 96 h. The optimal reaction time of the immobilized cells was determined by analyzing the changes in the trend of D-galactose conversion of the immobilized cells.

### 2.6. High Substrate Concentration Reaction and Recycle Measurement of Immobilized Cells

The D-galactose substrate solution of 150 g/L and 250 g/L were prepared. Free cells (100 g/L) and 0.5 g immobilized cells were added to the respective solutions to evaluate the D-tagatose production capacity of the immobilized cells under the optimum conditions. Then, free and immobilized cells were subjected to 10 consecutive batch reactions in 250 g/L D-galactose solution (pH 6.5) at 60 °C, with each cycle lasting 20 h. At the end of each cycle, the free and immobilized cells were washed with 10 mM acetate buffer (pH 6.5) to remove the residual substrate and then re-suspended in a fresh substrate solution of the same concentration. After each batch, 1 mL of the reaction mixture was collected to determine the titer of D-tagatose.

### 2.7. Analytical Methods

The quantification of D-galactose and D-tagatose was performed in a high-performance liquid chromatography system (HPLC, Shimadzu, Kyoto, Japan, LC-20AT) with a differential refractive index detector. Prior to analysis, samples were pre-filtered through a 0.22 μm sterile membrane filter to remove particulate matter. Chromatographic separation was achieved using a Hi-Plex Ca column (7.7 × 300 mm column, Agilent, Santa Clara, CA, USA). Temperatures of the column and the RID were maintained at 85 °C and 40 °C, respectively. Samples were injected and eluted with pure water at a flow rate of 0.6 mL/min. Corresponding standards solutions were run for the identification and quantification of the respective peak areas. All the experiments were performed in triplicate.

### 2.8. Statistical Analysis

One-way analysis of variance (ANOVA) and post-hoc multiple comparisons (Tukey’s post-hoc test) were used to determine significant differences between the experimental factors. Comparisons between two factors (e.g., two enzyme activities) were performed using *t*-tests. Each experiment was repeated three times, and all analyses were completed using GraphPad Prism 9.0 software and expressed as mean ± standard deviation (SD).

## 3. Results and Discussion

### 3.1. Optimization of Whole-Cell Reaction Conditions

**Figure 1:**
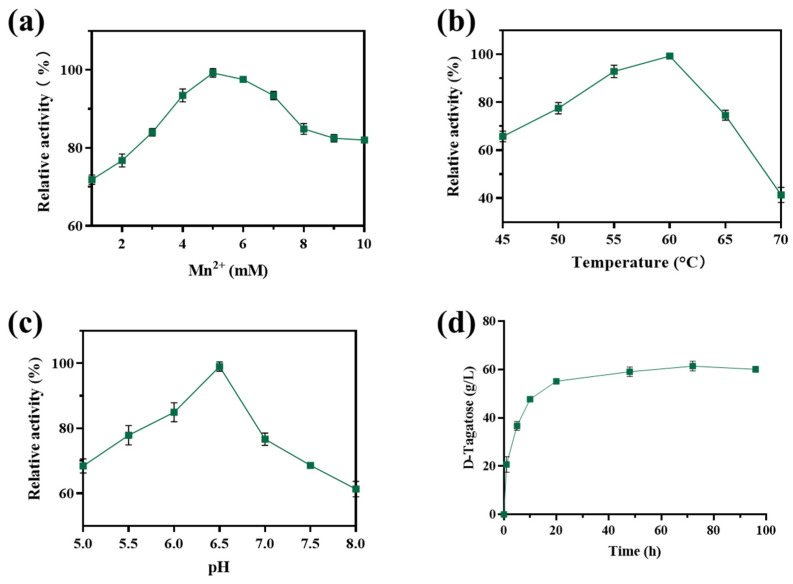
Effect of key reaction parameters on whole-cell viability of EC*araA*_F118M/F279I_. (**a**) The effect of Mn^2+^ concentrations. (**b**) The effect of temperature. (**c**) The effect of pH. (**d**) Whole-cell catalyzed production of D-tagatose from D-galactose.

The effects of varying concentrations of Mn^2+^ on the activity of whole cells were investigated. As shown in Figure 1a, the viability of whole cells at 45 °C was about 66%. Then, the whole-cell activity exhibited a significant increase with an increase in Mn^2+^ concentrations from 1 to 5 mM. There was a gradual decline with a further increase in Mn^2+^ concentration up to 10 mM. Therefore, 5 mM Mn^2+^ was selected as the optimal concentration for further experiments. The experimental results under various temperatures are presented in Figure 1b. The whole-cell activity was found to increase gradually with the increase in temperature, reaching its maximum at 60 °C. However, when the temperature was higher than 60 °C, the cell activity decreased sharply. Notably, whole cells demonstrated superior thermal tolerance compared to the purified LpAI enzyme, which has an optimal temperature of 50 °C, highlighting the advantages of whole-cell systems over purified enzymes for D-tagatose production [28]. The pH-dependent activity of whole cells was assessed at 60 °C with 5 mM Mn^2+^. As illustrated in Figure 1c, the whole-cell activity remained more than 80% relative activity under slightly acidic conditions (pH 6.0–6.5) but decreased at pH values above 6.5. Compared to the purified LpAI enzyme, which has an optimal pH of 5.0, the whole-cell system exhibited a higher optimal pH 6.5 while remaining within the acidic range [29]. At pH 5.0–8.0, the whole cells showed more than 60% viability, and the tolerance range was wide.

The conversion kinetics of D-galactose (150 g/L) by EC*araA*_F118M/F279I_ whole cells were analyzed under optimal conditions (60 °C, pH 6.5, 5 mM Mn^2+^), as shown in Figure 1d, the titer of D-tagatose increased gradually as the reaction time progressed up to 20 h when the D-tagatose titer reached 55.2 g/L. However, the highest substrate conversion rate was observed after 10 h. Equilibrium was achieved after 48 h, indicating that the stabilized within 20 h, which may represent the optimal time for maximizing production efficiency. In summary, the optimal conditions for whole-cell activity were determined to be 5 mM Mn^2+^, 60 °C, and pH 6.5.

### 3.2. Optimization of Immobilized Cell Preparation Conditions

#### 3.2.1. Effects of CaCl_2_ and Sodium Alginate Concentrations on Immobilized Cell Activity

**Figure 2:**
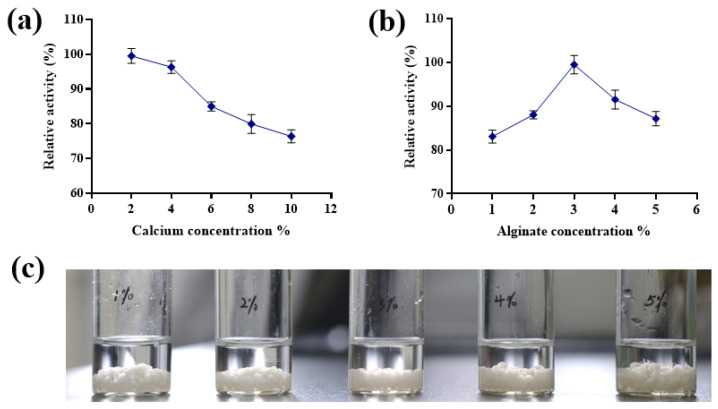
Effects of CaCl_2_ and sodium alginate concentrations on immobilized cell activity. (**a**) Effect of CaCl_2_ concentration on the viability of immobilized cells. (**b**) Effect of sodium alginate concentration on the viability of immobilized cells. (**c**) Immobilized microspheres with different concentrations of sodium alginate.

Ca^2+^ is the most widely used divalent cation in alginate immobilization. During gel formation with alginate, excessively high Ca^2+^ concentrations result in a densely packed gel structure with reduced pore size, hindering substrate accessibility to the immobilized cells/enzymes. Conversely, insufficient Ca^2+^ concentrations lead to weak gel rigidity, poor mechanical strength, and compromised structural stability of the immobilized system [30]. As shown in Figure 2a, the activity immobilized cells peaked at a CaCl_2_ concentration of 2%. Lower CaCl_2_ concentrations produced beads prepared with inadequate mechanical strength and irregular sizes, rendering them unsuitable for reliable measurements. With an increase in CaCl_2_ concentration, the viability of immobilized cells decreased gradually. Therefore, a 2% CaCl_2_ concentration was selected for follow-up experiments.

The sodium alginate concentration significantly influences the bond strength, swelling properties, and pore formation rate of the immobilized matrix, playing a critical role in cell/enzyme immobilization [31]. At low sodium alginate concentrations, the immobilized beads exhibit large pores, leading to cell leakage. Conversely, excessively high sodium alginate concentrations hinder substrate diffusion to the cells and complicate the bead formation process, resulting in irregular bead sizes, shapes, and tailing phenomena [32]. The effects of different concentrations of sodium alginate on the activity of immobilized cells are shown in Figure 2b. It can be seen that the activity of the immobilized cells increased as the sodium alginate concentration increased up to 3%. A further increase in the concentration of sodium alginate resulted in a gradual decline in the activity of the immobilized cells. Notably, at a sodium alginate concentration of 5%, the immobilized beads exhibited filamentous tailing (Figure 2c). Based on these findings, the optimal sodium alginate concentration for immobilization was found to be 3%.

#### 3.2.2. Effects of Hardening Time and Addition of Wet Cells on Immobilized Cell Activity

**Figure 3:**
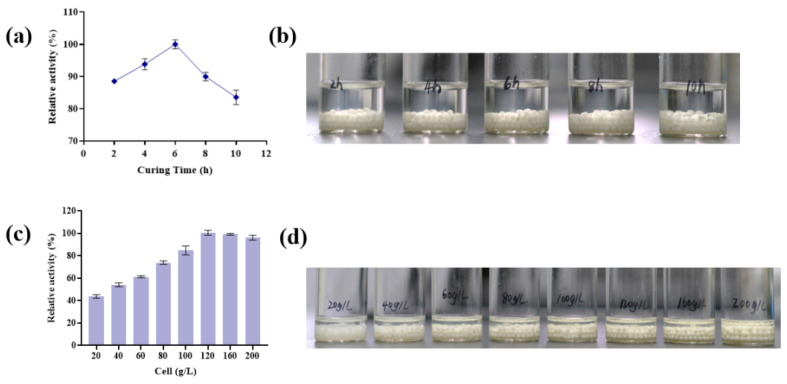
Effects of hardening time and addition of wet cells on immobilized cell activity. (**a**) Effects of immobilization time on the viability of immobilized cells under optimal conditions (60 °C, pH 6.5, 5 mM Mn^2+^). (**b**) Morphology of immobilized microspheres prepared with 100 g/L wet cells at pH 6.5 under different immobilization durations. (**c**) Effects of cell mass on the viability of immobilized cells under optimal conditions (60 °C, pH 6.5, 5 mM Mn^2+^). (**d**) Immobilized microspheres with different cell concentrations at pH 6.5.

Choosing an appropriate hardening time is crucial for enhancing the mechanical strength of immobilized beads. As shown in Figure 3a,b, immobilized cell activity reached 90% at a hardening time of 2 h. Insufficient hardening time may result in incomplete bead formation, resulting in cell leakage. As the hardening time increased, immobilized cell activity increased gradually, peaking at 6 h. Prolonging the hardening time beyond 6 h, caused a significant decline in activity. Excessive hardening time can lead to overly rigid beads, hindering substrate diffusion, while prolonged exposure to CaCl_2_ may damage internal cells and reduce enzyme activity, ultimately decreasing immobilized cell performance. The embedding capacity of cells within the sodium alginate matrix is limited. Excessive cell loading can lead to overcrowding, which reduces substrate accessibility and may result in cell leakage [33]. On the other hand, insufficient cell loading fails to fully utilize the internal space of the beads, leading to suboptimal enzymatic activity [34]. As illustrated in Figure 3c,d, the activity of immobilized cells increased with cell concentration ranging from 20–120 g/L but slightly decreased at concentrations exceeding 120 g/L. Therefore, a cell concentration of 120 g/L was identified as optimal for immobilization.

### 3.3. Effect of Reaction Condition and Stability of Immobilized Cells on Biocatalyst Activity

**Figure 4:**
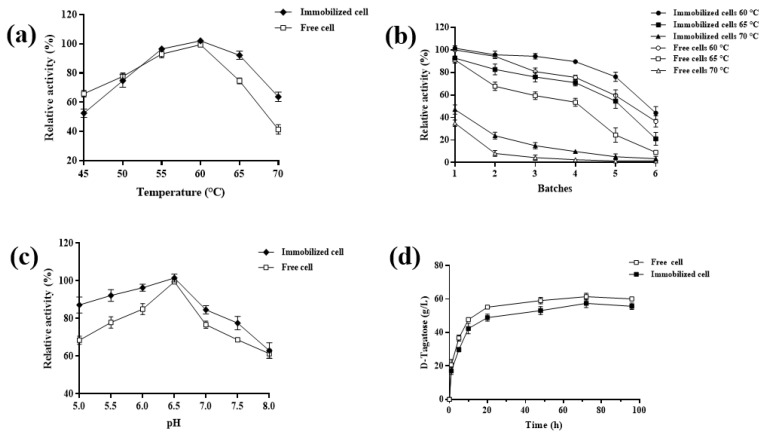
Optimization of reaction conditions and stability of immobilized cells. (**a**) Effects of temperature on immobilized cell activity. (**b**) Thermal stability of free and immobilized cells. (**c**) Effects of pH on immobilized cell activity. (**d**) Free and immobilized cells catalyzed the production of D-tagatose from D-galactose.

The effects of temperature on the activity of immobilized cells and comparison with free cells is shown in Figure 4a. Both free cells and immobilized cells exhibited optimal activity at 60 °C. At temperature below 50 °C, free cells demonstrated higher activity compared to immobilized cells. However, at temperatures above 60 °C, the relative activity of free cells declined rapidly, while immobilized cells maintained robust activity. These results indicate that the immobilized cells possess superior thermal stability and have a wider temperature range than free cells. A key objective of immobilization is to enhance the stability of cells/enzymes. To evaluate this, the activity of immobilized cells was measured over six consecutive reaction batches at different temperatures and compared with that of free cells, as shown in Figure 4b. At 60 °C, the activity of free cells and immobilized cells was comparable during the first two batches. Beyond the second batch, the activity of free cells decreased significantly, remaining approximately 10% lower than that of immobilized cells. At temperatures above 65 °C, immobilized cells consistently outperformed free cells in each batch. Notably, after four batches at 70 °C, free cells retained only 2.5% of their initial activity, while immobilized cells maintained 10% activity. The enhanced stability of immobilized cells can be attributed to reduced conformational flexibility and the protective microenvironment provided by alginate hydrogel carriers. These factors enable immobilized cells to retain the functional characteristics of free cells while exhibiting significantly improved stability [35].

The effect of pH on the activity of immobilized cells was determined together and results were compared with those of free cells. As shown in Figure 4c, both free cells and immobilized cells exhibited optimal activity at pH 6.5. It is worth noting that the immobilized cells showed stronger tolerance to pH values, and the relative activity of almost all pH values (especially under acidic conditions) was higher than that of free cells. In a word, the optimum pH value of immobilized cells was 6.5, and it could maintain good vitality under acidic conditions, which was better than that of free cells. These findings align with the results reported by Zheng et al. [35]. Subsequently, the conversion kinetics of immobilized cells were investigated using 150 g/L D-galactose under the optimal conditions (50 °C, pH 6.5) over 96 h, with results compared to those of free cells. As illustrated in Figure 4d, the substrate conversion trend of immobilized cells was consistent with that of free cells, although the D-tagatose titer provided by immobilized cells (57.5 g/L) was slightly lower than that produced by the free cells (61.4 g/L). For both immobilized and free cells, it was observed that the rate of transformation decreased dramatically after 20 h until 100 h when it increased by less than 10%, and a single batch reaction time of 20 h was chosen for subsequent experiments, considering the most economical approach to production costs.

The above results showed that the immobilized cells demonstrated significantly enhanced stability under industrially challenging conditions compared to their free-cell counterparts. While free cells exhibited rapid activity loss at temperatures exceeding 60 °C and under extreme pH conditions, the immobilized system maintained remarkable thermal stability at elevated temperatures and showed superior tolerance in acidic environments (pH 5.0–6.5). This robust performance, together with the system’s sustained productivity across multiple operational batches as shown in Figure 4b, clearly demonstrates its exceptional suitability for industrial applications where temperature and pH fluctuations frequently occur. The combination of these protective effects resulted in a highly stable biocatalyst capable of maintaining activity under harsh process conditions that would normally lead to rapid deactivation of non-immobilized cells.

### 3.4. D-Tagatose Production Using Immobilized Cells Under a Higher Substrate Concentration

**Figure 5:**
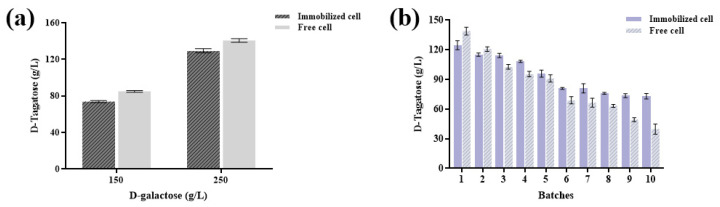
D-tagatose production using immobilized cells. (**a**) The yield of D-tagatose at two different substrate concentrations. (**b**) Stability of immobilized cells over cycles.

In order to obtain a higher concentration of D-tagatose, the substrate concentration was increased to 250 g/L and compared with the results obtained from 150 g/L D-galactose, as shown in Figure 5a. At D-galactose concentrations of 150 g/L and 250 g/L, the immobilized cells provided a D-tagatose titer of 76.00 g/L and 129.43 g/L, respectively. However, the titers were found to be lower than the titers provided by the free cells under similar conditions, as free cells produced 84.95 g/L and 140.66 g/L D-tagatose at 150 g/L and 250 g/L D-galactose concentrations, respectively. It can be seen that increasing the concentration of D-galactose substrate significantly enhances D-tagatose production; thus, this higher concentration was subsequently applied in future experiments.

The stability and reusability of immobilized cells are critical for industrial applications and represent a key objective of immobilized cell systems [36]. Immobilized and free cells were tested over 10 consecutive reaction batches in 250 g/L D-galactose solution, As presented in Figure 5b, the immobilized cells exhibited lower yield loss compared to free cells, with initial D-tagatose titer and productivity of 124.77 g/L and 6.24 g/L/h, respectively with immobilized cells, which were 140.00 g/L and 7.00 g/L/h with free cells. After six batches, the D-tagatose titer with free cells decreased significantly (*p* < 0.05), which further declined to 28.7% of the initial D-tagatose after the 10th batch, with the productivity decreasing to 1.99 g/L/h. In contrast, immobilized cells retained 58.6% titer (73.11 g/L) with higher productivity (3.66 g/L/h) after the 10th batch, demonstrating superior stability during reuse. Over the 10 batches, the average D-tagatose titer and productivity for immobilized cells were 94.34 g/L and 4.72 g/L, respectively. Although the activity of immobilized cells gradually decreased with repeated use, their performance remained significantly higher than that of free cells. Specifically, the D-tagatose yield of immobilized cells in the 10th batch was 83.6% higher than that of free cells (73.11 g/L vs. 39.83 g/L), and the residual activity of immobilized cells was markedly superior (Figure 5b). The above results demonstrate the immobilized system’s superior kinetic stability, where despite a slightly lower initial productivity (6.24 vs. 7.00 g/L/h), the immobilized cells maintained significantly higher sustained activity, retaining 58.6% of initial titer versus just 28.7% for free cells after 10 batches, ultimately achieving 83.6% greater final titer (73.11 vs. 39.83 g/L). This robust performance under repeated use suggests the alginate matrix effectively preserves enzyme conformation (V_max_-related stability) while maintaining favorable substrate accessibility (K_m_-related efficiency) at high industrial substrate loads (250 g/L), making it particularly suitable for continuous production processes where long-term catalyst performance is paramount. In summary, the use of immobilized EC*araA*_F118M/F279I_ whole cell as a catalyst for D-tagatose production demonstrated excellent stability, offering a promising approach for simple, cost-effective, and sustainable industrial-scale D-tagatose production.

## 4. Conclusions

This study developed an innovative immobilized whole-cell biocatalytic system for efficient D-tagatose production through three key advancements: (1) the creation of a thermostable biocatalyst by combining the engineered EC*araA*/_F118M/F279I_ strain with optimized sodium alginate immobilization (3% alginate, 2% CaCl_2_, 120 g/L cells); (2) significant stability improvements under industrial conditions, maintaining 76% activity after 5 batches at 60 °C and showing 83.6% higher productivity than free cells after 10 batches; and (3) the demonstration of high-yield conversion at elevated substrate loading (124.77 g/L titer at 250 g/L D-galactose). The immobilized system’s unique combination of thermal resilience (functional up to 70 °C), pH tolerance (active at pH 5.0–8.0), and operational durability represents a breakthrough for continuous D-tagatose manufacturing, addressing key limitations of conventional free-cell bioprocesses. These findings establish a reference for stable, cost-effective biocatalysis in industrial sugar isomerization applications.

## Data Availability

The original contributions presented in the study are included in the article. Further inquiries can be directed to the corresponding authors.
